# Synthesis, bioactivity, and molecular docking studies: novel arylpiperazine derivatives as potential new-resistant AR antagonists

**DOI:** 10.3389/fchem.2025.1557275

**Published:** 2025-03-28

**Authors:** Hua Jiang, Haowei Chen, Ya Wang, Huaxin Xu, Hong Chen

**Affiliations:** ^1^ Department of Urology, The Fifth Affiliated Hospital of Zunyi Medical University (Zhuhai Sixth People’s Hospital), Zhuhai, China; ^2^ Luoyang Key Laboratory of Organic Functional Molecules, College of Food and Drug, Luoyang Normal University, Luoyang, China

**Keywords:** prostate cancer, antagonistic activity, binding affinities, docking study, AR antagonists

## Abstract

The majority of patients with androgen-dependent prostate cancer (PCa) develop resistance to hormone therapy after approximately 18–24 months of androgen deprivation therapy treatment. During this process, PCa cells progressively lose their sensitivity to androgens and evolve into castration-resistant prostate cancer leading to uncontrolled tumor growth and ultimately the failure of endocrine therapy. To develop potential anti-prostate cancer agents, in this study, we identified a novel ether-type arylpiperazine derivative as a potent androgen receptor (AR) antagonist, uncovering a series of effective antiproliferative compounds. The derivatives (**7, 11, 17, 19, 20, 21, 22, 23**, and **24**) demonstrated strong cytotoxicity against cancer cells, with **17, 19, 20**, and **23** showing significant androgen receptor antagonistic activity (Inhibition% >60) and robust AR binding affinities. The structure-activity relationship (SAR) of these developed derivatives was discussed based on data. Docking study suggested that the compound **19** mainly bind to AR ligand binding pocket site through Van der Waals’ force interactions. This research presents a promising lead compound for developing anticancer agents targeting prostate cancer therapy.

## Highlights

 • A series of arylpiperazine derivative was synthesized.
 • The anti-prostate cancer activities of derivatives were investigated.
 • Binding affinity and antagonistic potency of derivatives were also investigated against AR.
 • Some derivatives exhibited strong activities against AR and cancer cells.
 • Molecular docking and SAR of derivatives were also studied.

## Introduction

According to the National Cancer Center of China’s 2024 National Cancer Report, there has been an increasing trend in both the incidence and mortality rates of prostate cancer in China in recent years, ranking sixth among the top ten malignant tumors in men (Zheng et al., 2024). The 2022 American Cancer Statistics Report estimated that prostate cancer would be the most common newly diagnosed cancer, accounting for 27%, and the second leading cause of cancer death, accounting for 11% (Siegel et al., 2022). The growth of prostate cancer cells depends on androgens, which exert their biological functions through the AR signaling pathway. Abnormal activation of the AR signaling pathway is the fundamental reason for the occurrence and development of prostate cancer (Dai et al., 2023; He et al., 2022; Jamroze et al., 2021).

Early-stage localized prostate cancer can be completely cured through surgical treatment and radiation therapy. For non-localized, inoperable prostate cancer patients, the first-line therapy is ADT (Choi et al., 2022). Endocrine therapy, while effective at controlling the progression of prostate cancer during the initial stages of treatment, leads to almost all initially hormone-sensitive tumors transforming into CRPC after 18–24 months of therapy (Vellky and Ricke, 2020). This presents significant clinical challenges, as there is currently no effective treatment regimen available for CRPC. Currently, there is no effective treatment for CRPC, although its molecular mechanisms of occurrence and development have not been fully elucidated, extensive research has found that 80% of advanced CRPC overexpress AR (Formaggio et al., 2021; Visakorpi et al., 1995), and the expression of AR in bone metastases is higher than in primary tumors (Fontana et al., 2022; Lu et al., 2020; Obinata et al., 2020). The application of next-generation ADTs (such as enzalutamide and abiraterone) can suppress the progression of CRPC by inhibiting AR in CRPC cells, and the absence of AR in CRPC cells can lead to cell death. This phenomenon indicates that the survival and growth of CRPC still depend on the AR signaling pathway, and the reactivation of AR is the fundamental cause of CRPC. Patients with CRPC constitute the main population at risk of dying from prostate cancer. Therefore, AR has become an important target for the treatment of prostate cancer. However, the development of resistance is a common issue in current endocrine therapies for prostate cancer (Schmidt et al., 2021).

**FIGURE 1:**
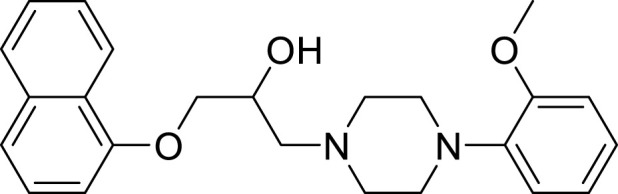
Structures of naftopidil.

Thus, finding and developing highly effective AR-targeted antagonists that combat resistance for the endocrine treatment of prostate cancer is an urgent need. Naftopidil (NAF, Figure 1), a class of arylpiperazine derivatives, selectively blocks the α1a/1d receptor subtypes, reduces the levels of dihydrotestosterone within the prostate tissue and cells, promotes apoptosis, and is currently used in the treatment of benign prostatic hyperplasia (Zhan et al., 2022). Furthermore, studies have found that NAF can effectively inhibit the proliferation of prostate cancer cells PC-3 and LNCaP, inducing apoptosis (Ishii et al., 2018; Iwamoto et al., 2017; Maesaka et al., 2021). Kinoyama et al. (2005), Kinoyama et al. (2004) reported that arylpiperazine derivatives exhibit significant AR antagonistic activity, with an IC_50_ of 0.11 μmol/L, compared to bicalutamide’s IC_50_ of 50 μmol/L. They can inhibit prostate hyperplasia without affecting serum testosterone levels. In recent years, our research team has conducted extensive preliminary basic research on the anti-prostate cancer activity of arylpiperazine derivatives, discovering that some arylpiperazine derivatives show good cytotoxic activity (Chen et al., 2018a; Chen et al., 2018b; Chen et al., 2017; Chen et al., 2015; Chen et al., 2019a; Qi et al., 2022a) and exhibit better antagonistic activity and affinity for AR (Chen et al., 2019a; Chen et al., 2019b).

**SCHEME 1:**
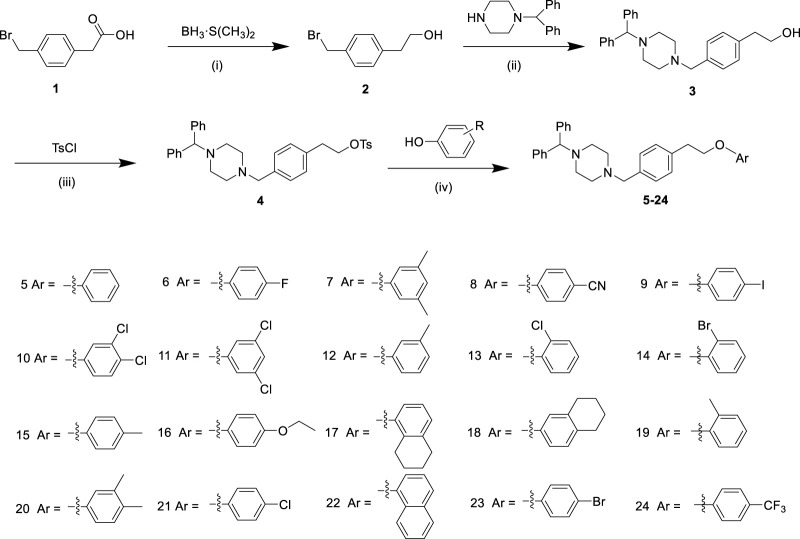
The synthesis route of derivatives **5–24**. Reagents and conditions: (i) BH_3_·S(CH_3_)_2_, THF, 0°C for 1 h, and then room temperature for 12 h; (ii) 1-(Diphenylmethyl) piperazine, K_2_CO_3_, CH_3_CN, reflux, 12 h; (iii) TsCl, Et_3_N and 4-dimethylaminopyridine, Cl_2_CH_2_, 0°C, 16 h; (iv) Phenol, K_2_CO_3_, CH_3_CN, reflux, 12 h.

Although the reported arylpiperazine derivatives possess significant AR antagonistic activity, there is less research on their resistance evaluation and antitumor molecular mechanisms. Based on the aforementioned studies, the drug design strategy of this project is to design and synthesize a new class of arylpiperazine derivatives based on naftopidil (Scheme 1) on the foundation of previous research. The aim is to investigate their biological activity, resistance, and antitumor molecular mechanisms, thereby obtaining new drugs with stronger antagonistic activity and resistance to treat prostate cancer.

## Materials and methods

### General chemistry

Reagents and solvents were procured via commercial channels. Organic solvents underwent distillation before use. Melting points were determined using an uncalibrated SGW X-4 micro melting point apparatus. NMR spectra were acquired on a Bruker AVANCE-400 spectrometer in CDCl_3_, employing TMS as an internal standard, with chemical shifts reported in δ (ppm) and coupling constants in Hertz. HRMS spectra were documented on an AB Sciex X500R QTOF mass spectrometer (Foster City, CA, United States). HPLC chromatogram was performed on UltiMate 3000 with H_2_O and CH_3_CN as the mobile phase. The completion of all reactions was monitored by thin-layer chromatography (TLC) performed on pre-coated silica gel 60 F_254_ TLC plates (VWR), with observations made under ultraviolet light at wavelengths of 254 and/or 365 nm (Sun et al., 2022; Sun et al., 2025).

### Cell lines

The cell lines PC-3, LNCaP, DU145 and WPMY-1 were purchased from the Cell Bank of the Chinese Academy of Sciences.

### Synthesis of 2-(4-(bromomethyl)phenyl)ethanol (2)

Compound **2** was synthesized using methods reported previously in the literature (Chen et al., 2018a; Chen et al., 2014).

### 2-(4-((4-benzhydrylpiperazin-1-yl)methyl)phenyl)ethan-1-ol (3)

Compound **3** was synthesized using methods reported previously in the literature (Chen et al., 2018b), and sesamol was substituted with 1-(diphenylmethyl)-piperazin. White solid (ethyl acetate). Yield: 70% from compound 1; M.p. 101.4°C–101.8°C; ^1^H NMR (400 MHz, CDCl3): δ 7.38 (d, *J* = 7.4 Hz, 4H), 7.25–7.20 (m, 6H), 7.13 (t, *J* = 8.6 Hz, 4H), 4.21 (s, 1H), 3.77 (t, *J* = 6.7 Hz, 2H), 3.46 (s, 2H), 2.80 (t, *J* = 6.6 Hz, 2H), 2.45 (s, 8H). HRMS (ESI) m/z [M + H]^+^: calcd for C_26_H_31_N_2_O: 387.2431, found: 387.2448.

### 4-((4-benzhydrylpiperazin-1-yl)methyl)phenethyl 4-methylbenzenesulfonate (4)

Compound **4** was synthesized using methods reported previously in the literature (Chen et al., 2018b). White solid (ethyl acetate). M.p. 106.3°C–106.7°C; Yield, 87%. ^1^H NMR (400 MHz, CDCl_3_): *δ* 7.68 (d, *J* = 8.2 Hz, 2H), 7.39 (d, *J* = 7.4 Hz, 4H), 7.24 (t, *J* = 7.8 Hz, 6H), 7.19–7.12 (m, 4H), 7.02 (d, *J* = 7.8 Hz, 2H), 4.22 (s, 1H), 4.17 (t, *J* = 7.1 Hz, 2H), 3.48 (s, 2H), 2.90 (t, *J* = 7.1 Hz, 2H), 2.46 (s, 8H), 2.38 (s, 3H). HRMS (ESI) m/z [M + H]^+^: calcd for C_33_H_37_N_2_O_3_S: 541.2519, found: 541.2601.

### General procedure for the preparation of arylpiperazine derivative 5-24

Phenol (0.27 mmol, 1.5 equiv) and potassium carbonate (1.08 mmol, 6.0 equiv) were added to a solution of 4-((4-benzhydrylpiperazin-1-yl)methyl)phenethyl 4-methylbenzenesulfonate **4** (0.18 mmol, 1.0 equiv) in acetonitrile (CH_3_CN, 15 mL). The reaction mixture was heated to 85°C and stirred for 12 h. Afterward, the mixture was cooled down to room temperature. The reaction mixture was then filtered, and the filtrate was concentrated under vacuum. The residue was purified by silica gel column chromatography using a petroleum ether:ethyl acetate ratio of 25:1 (v/v) to obtain the corresponding product (**5**–**24**).

### Experimental data of 1-benzhydryl-4-(4-(2-phenoxyethyl)benzyl)piperazine (5)

White solid (ethyl acetate); M.p. 96.3°C–96.8°C; Yield, 82%. The purity = 98.5%. ^1^H NMR (400 MHz, CDCl_3_): *δ* 7.39 (d, *J* = 7.6 Hz, 4H), 7.27 (s, 1H), 7.25 (d, *J* = 2.3 Hz, 2H), 7.23 (d, *J* = 7.0 Hz, 5H), 7.19 (d, *J* = 8.0 Hz, 2H), 7.14 (t, *J* = 7.2 Hz, 2H), 6.92 (d, *J* = 7.3 Hz, 1H), 6.88 (d, *J* = 8.2 Hz, 2H), 4.22 (s, 1H), 4.13 (t, *J* = 7.1 Hz, 2H), 3.48 (s, 2H), 3.05 (t, *J* = 7.1 Hz, 2H), 2.46 (s, 4H), 2.40 (s, 4H). ^13^C NMR (100 MHz, CDCl_3_): *δ* 158.8, 142.8, 137.0, 136.2, 129.5, 128.8, 128.4, 128.0, 126.9, 120.7, 114.6, 76.2, 68.6, 62.8, 53.4, 51.9, 35.5. HRMS (ESI) m/z [M + H]^+^: calcd for C_32_H_35_N_2_O: 463.2744, found: 463.2745.

### Experimental data of 1-benzhydryl-4-(4-(2-(4-fluorophenoxy)ethyl)benzyl)piperazine (6)

Light yellow solid (ethyl acetate); M.p. 90.1°C–90.4°C; Yield, 78%. The purity = 98.3%. ^1^H NMR (400 MHz, CDCl_3_): *δ* 7.30 (d, *J* = 7.4 Hz, 4H), 7.15 (t, *J* = 7.2 Hz, 6H), 7.10 (d, *J* = 7.7 Hz, 2H), 7.06 (t, *J* = 7.3 Hz, 2H), 6.84 (t, *J* = 8.3 Hz, 2H), 6.72–6.69 (m, 2H), 4.14 (s, 1H), 3.99 (t, *J* = 7.0 Hz, 2H), 3.41 (s, 2H), 2.94 (t, *J* = 6.9 Hz, 2H), 2.39 (s, 4H), 2.32 (s, 4H). ^13^C NMR (100 MHz, CDCl_3_): *δ* 158.5, 155.5 (d, *J* = 112.5 Hz), 142.8, 137.0, 136.0, 129.5, 128.8, 128.5, 128.0, 126.9, 115.8 (d, *J* = 22.9 Hz), 115.6 (d, *J* = 8.0 Hz), 76.2, 69.4, 62.7, 53.3, 51.8, 35.5. HRMS (ESI) m/z [M + H]^+^: calcd for C_32_H_34_FN_2_O: 481.2650, found: 481.2647.

### Experimental data of 1-benzhydryl-4-(4-(2-(3,5-dimethylphenoxy)ethyl)benzyl)piperazine (7)

White solid (ethyl acetate); M.p. 123.2°C–123.5°C; Yield, 78%. The purity = 98%. ^1^H NMR (400 MHz, CDCl_3_): *δ* 7.39–7.37 (m, 4H), 7.21–7.18 (m, 8H), 7.12 (d, *J* = 7.0 Hz, 2H), 6.65–6.50 (m, 3H), 4.20 (s, 1H), 4.07 (t, *J* = 7.0 Hz, 2H), 3.46 (s, 2H), 3.01 (d, *J* = 6.6 Hz, 2H), 2.44 (s, 4H), 2.38 (s, 4H), 2.20 (s, 3H), 2.23 (s, 3H). ^13^C NMR (100 MHz, CDCl_3_): *δ* 159.0, 142.9, 139.2, 137.2, 136.2, 129.5, 128.5, 128.1, 127.0, 122.6, 122.5, 76.3, 68.6, 62.9, 53.4, 52.0, 35.7, 21.6. HRMS (ESI) m/z [M + H]^+^: calcd for C_34_H_39_N_2_O: 491.3057, found: 491.3059.

### Experimental data of 4-(4-((4-benzhydrylpiperazin-1-yl)methyl)phenethoxy)benzonitrile (8)

White solid (ethyl acetate); M.p. 112.3°C–112.7°C; Yield, 51%. The purity = 98.7%. ^1^H NMR (400 MHz, CDCl_3_): *δ* 7.53 (d, *J* = 8.6 Hz, 2H), 7.39 (d, *J* = 7.4 Hz, 4H), 7.24 (t, *J* = 7.3 Hz, 6H), 7.19–7.13 (m, 4H),6.90 (d, *J* = 8.6 Hz, 2H), 4.22 (s, 1H), 4.16 (t, *J* = 7.0 Hz, 2H), 3.49 (s, 2H), 3.07 (t, *J* = 6.9 Hz, 2H), 2.46 (s, 4H), 2.40 (s, 4H). ^13^C NMR (100 MHz, CDCl3): δ 162.1, 142.8, 136.6, 136.3, 134.0, 129.5, 128.8, 128.4, 128.0, 126.9, 119.2, 115.2, 104.0, 76.2, 69.0, 62.7, 53.4, 51.9, 35.2. HRMS (ESI) m/z [M + H]^+^: calcd for C_33_H_34_N_3_O: 488.2696, found: 488.2714.

### Experimental data of 1-benzhydryl-4-(4-(2-(4-iodophenoxy)ethyl)benzyl)piperazine (9)

White solid (ethyl acetate); M.p. 113.4°C–113.9°C; Yield, 76%. The purity = 98.7%. ^1^H NMR (400 MHz, CDCl_3_): δ 7.51 (d, *J* = 8.8 Hz, 2H), 7.39 (d, *J* = 7.5 Hz, 4H), 7.26 (s, 1H), 7.23 (d, *J* = 7.0 Hz, 5H), 7.18–7.13 (m, 4H), 6.64 (d, *J* = 8.8 Hz, 2H), 4.22 (s, 1H), 4.08 (t, *J* = 7.1 Hz, 2H), 3.49 (s, 2H), 3.03 (t, *J* = 7.0 Hz, 2H), 2.47 (s, 4H), 2.42 (s, 4H). ^13^C NMR (100 MHz, CDCl_3_): δ 158.7, 142.8, 138.2, 136.7, 136.2, 129.5, 128.8, 128.4, 128.0, 126.9, 117.0, 82.7, 76.2, 68.8, 62.7, 53.3, 51.8, 35.3. HRMS (ESI) m/z [M + H]^+^: calcd for C_32_H_34_IN_2_O: 589.1710, found: 589.1740.

### Experimental data of 1-benzhydryl-4-(4-(2-(3,4-dichlorophenoxy)ethyl)benzyl)piperazine (10)

Light yellow solid (ethyl acetate); M.p. 130.7°C–131.2°C; Yield, 68%. The purity = 98.8%. ^1^H NMR (400 MHz, CDCl_3_): δ 7.31 (d, *J* = 7.1 Hz, 4H), 7.19–7.13 (m, 7H), 7.09–7.04 (m, 4H), 6.86 (d, *J* = 2.7 Hz, 1H), 6.61 (dd, *J* = 8.8 Hz, *J* = 2.6 Hz, 1H), 4.13 (s, 1H), 3.99 (t, *J* = 7.0 Hz, 2H), 3.40 (s, 2H), 2.94 (t, *J* = 6.9 Hz, 2H), 2.37 (s, 4H), 2.31 (s, 4H). ^13^C NMR (100 MHz, CDCl3): δ 156.8, 141.7, 135.5, 135.4, 131.8, 129.6, 128.4, 127.7, 127.4, 126.9, 125.8, 122.8, 115.3, 113.5, 75.2, 68.2, 61.7, 52.3, 50.8, 34.2. HRMS (ESI) m/z [M + H]^+^: calcd for C_32_H_33_Cl_2_N_2_O: 531.1965, found: 531.1991.

### Experimental data of 1-benzhydryl-4-(4-(2-(3,5-dichlorophenoxy)ethyl)benzyl)piperazine (11)

Light yellow solid (ethyl acetate); M.p. 129.5°C–130.2°C; Yield, 77%. The purity = 97.9%. ^1^H NMR (400 MHz, CDCl_3_): *δ* 7.30 (d, *J* = 7.5 Hz, 4H), 7.14 (d, *J* = 6.6 Hz, 6H), 7.08–7.04 (m, 4H), 6.83 (s, 1H), 6.67 (s, 2H), 4.14 (s, 1H), 3.99 (t, *J* = 6.8 Hz, 2H), 3.40 (s, 2H), 2.93 (t, *J* = 6.8 Hz, 2H), 2.37 (s, 4H), 2.31 (s, 4H). ^13^C NMR (100 MHz, CDCl_3_): *δ* 159.9, 142.8, 136.6, 136.3, 135.4, 129.5, 128.8, 128.4, 128.0, 126.9, 121.0, 113.7, 76.3, 69.3, 62.8, 53.4, 51.9, 35.2. HRMS (ESI) m/z [M + H]^+^: calcd for C_32_H_33_Cl_2_N_2_O: 531.1965, found: 531.1985.

### Experimental data of 1-benzhydryl-4-(4-(2-(m-tolyloxy)ethyl)benzyl)piperazine (12)

White solid (ethyl acetate); M.p. 125.1°C–125.6°C; Yield, 80%. The purity = 98.8%. ^1^H NMR (500 MHz, CDCl_3_): *δ* 7.45 (d, *J* = 7.5 Hz, 4H), 7.31 (s, 1H), 7.30 (d, *J* = 7.7 Hz, 5H), 7.26 (d, *J* = 8.0 Hz, 2H), 7.20 (dd, *J* = 15.0 Hz, *J* = 7.3 Hz, 3H), 6.79 (d, *J* = 7.5 Hz, 1H), 6.76–6.74 (m, 2H), 4.28 (s, 1H), 4.18 (t, *J* = 7.2 Hz, 2H), 3.55 (s, 2H), 3.11 (t, *J* = 7.1 Hz, 2H), 2.53 (s, 4H), 2.47 (s, 4H), 2.36 (s, 3H). ^13^C NMR (125 MHz, CDCl_3_): *δ* 158.9, 142.8, 139.5, 137.1, 136.1, 129.5, 129.2, 128.9, 128.5, 128.0, 126.9, 121.6, 115.5, 111.5, 76.2, 68.6, 62.7, 53.3, 51.9, 35.5, 21.6. HRMS (ESI) m/z [M + H]^+^: calcd for C_33_H_37_N_2_O: 477.2900, found: 477.2972.

### Experimental data of 1-benzhydryl-4-(4-(2-(2-chlorophenoxy)ethyl)benzyl)piperazine (13)

White solid (ethyl acetate); M.p. 128.2°C–128.8°C; Yield, 74%. The purity = 98.2%. ^1^H NMR (400 MHz, CDCl_3_): *δ* 7.39 (d, *J* = 7.5 Hz, 4H), 7.33 (d, *J* = 7.3 Hz, 1H), 7.24 (t, *J* = 8.0 Hz, 8H), 7.15 (t, *J* = 6.9 Hz, 3H), 6.86 (d, *J* = 7.8 Hz, 2H), 4.22 (s, 1H), 4.18 (t, *J* = 7.0 Hz, 2H), 3.49 (s, 2H), 3.11 (t, *J* = 7.0 Hz, 2H), 2.46 (s, 4H), 2.39 (s, 4H). ^13^C NMR (100 MHz, CDCl_3_): *δ* 154.4, 142.8, 136.7, 136.3, 130.3, 129.4, 129.0, 128.4, 128.0, 127.6, 126.9, 123.0, 121.3, 113.4, 76.2, 69.8, 62.8, 53.3, 51.9, 35.4. HRMS (ESI) m/z [M + H]^+^: calcd for C_32_H_34_ClN_2_O: 497.2354, found: 497.2382.

### Experimental data of 1-benzhydryl-4-(4-(2-(2-bromophenoxy)ethyl)benzyl)piperazine (14)

White solid (ethyl acetate); M.p. 132.1°C–132.6°C; Yield, 81%. The purity = 98.5%. ^1^H NMR (400 MHz, CDCl_3_): *δ* 7.49 (d, *J* = 7.7 Hz, 1H), 7.38 (d, *J* = 7.4 Hz, 4H), 7.22 (d, *J* = 9.7 Hz, 8H), 7.18–7.12 (m, 3H), 6.81–6.75 (m, 2H), 4.21 (s, 1H), 4.14 (t, *J* = 6.8 Hz, 2H), 3.48 (s, 2H), 3.10 (t, J = 6.8 Hz, 2H), 2.46 (s, 4H), 2.40 (s, 4H). ^13^C NMR (100 MHz, CDCl_3_): *δ* 155.3, 142.8, 136.8, 136.2, 133.4, 129.5, 129.1, 128.5, 128.4, 128.0, 126.9, 121.9, 113.2, 112.3, 76.3, 69.9, 62.8, 53.4, 51.9, 35.5. HRMS (ESI) m/z [M + H]^+^: calcd for C_32_H_34_BrN_2_O: 541.1849, found: 541.1878.

### Experimental data of 1-benzhydryl-4-(4-(2-(p-tolyloxy)ethyl)benzyl)piperazine (15)

White solid (ethyl acetate); M.p. 126.4°C–126.9°C; Yield, 72%. The purity = 98.3%. ^1^H NMR (400 MHz, CDCl_3_): *δ* 7.39 (d, *J* = 7.4 Hz, 4H), 7.26–7.20 (m, 8H), 7.17 (t, *J* = 5.9 Hz, 2H), 7.05 (d, *J* = 8.3 Hz, 2H), 6.78 (d, *J* = 8.4 Hz, 2H), 4.22 (s, 1H), 4.11 (t, *J* = 7.2 Hz, 2H), 3.48 (s, 2H), 3.04 (t, *J* = 7.1 Hz, 2H), 2.46 (s, 4H), 2.40 (s, 4H), 2.26 (s, 3H). ^13^C NMR (100 MHz, CDCl_3_): *δ* 156.7, 142.8, 137.0, 136.1, 129.9, 129.4, 128.8, 128.4, 128.0, 126.9, 117.9, 114.5, 76.2, 68.8, 62.8, 53.3, 51.9, 35.5, 20.5. HRMS (ESI) m/z [M + H]^+^: calcd for C_33_H_37_N_2_O: 477.2900, found: 477.2915.

### Experimental data of 1-benzhydryl-4-(4-(2-(4-ethoxyphenoxy)ethyl)benzyl)piperazine (16)

White solid (ethyl acetate); M.p. 124.7°C–125.2°C; Yield, 67%. The purity = 98%. ^1^H NMR (400 MHz, CDCl_3_): *δ* 7.39 (d, *J* = 7.4 Hz, 4H), 7.26 (s, 1H), 7.23 (d, *J* = 7.9 Hz, 5H), 7.19 (d, *J* = 8.0 Hz, 2H), 7.14 (t, *J* = 7.4 Hz, 2H), 6.80 (s, 4H), 4.22 (s, 1H), 4.08 (t, *J* = 7.0 Hz, 2H), 3.95 (q, *J* = 13.8 Hz, *J* = 6.9 Hz, 2H), 3.50 (s, 2H), 3.03 (t, *J* = 7.0 Hz, 2H), 2.47 (s, 4H), 2.40 (s, 4H), 1.37 (t, *J* = 6.9 Hz, 3H). ^13^C NMR (100 MHz, CDCl_3_): *δ* 153.2, 152.9, 142.8, 137.1, 135.9, 129.5, 128.8, 128.4, 128.0, 126.9, 115.6, 115.4, 76.2, 69.4, 64.0, 62.7, 53.3, 51.8, 35.6, 15.0. HRMS (ESI) m/z [M + H]^+^: calcd for C_34_H_39_N_2_O_2_: 507.3006, found: 507.3019.

### Experimental data of 1-benzhydryl-4-(4-(2-((5,6,7,8-tetrahydronaphthalen-1-yl)oxy)ethyl)benzyl)piperazine (17)

White solid (ethyl acetate); M.p. 115.8°C–116.3°C; Yield, 66%. The purity = 98.8%. ^1^H NMR (400 MHz, CDCl_3_): *δ* 7.68 (d, *J* = 8.0 Hz, 2H), 7.39 (d, *J* = 7.6 Hz, 4H), 7.27–7.22 (m, 5H), 7.17 (t, *J* = 7.6 Hz, 4H), 7.03 (d, *J* = 7.7 Hz, 2H), 4.22 (s, 1H), 4.17 (t, *J* = 7.1 Hz, 2H), 3.47 (s, 2H), 2.91 (t, *J* = 7.1 Hz, 2H), 2.45–2.40 (m, 12H), 1.42–1.28 (m, 4H). ^13^C NMR (100 MHz, CDCl_3_): *δ* 144.6, 142.8, 136.7, 134.9, 133.1, 129.8, 129.5, 128.7, 128.4, 128.0, 127.8, 126.9, 76.2, 70.6, 62.6, 53.3, 51.9, 35.0, 21.6. HRMS (ESI) m/z [M + H]^+^: calcd for C_36_H_41_N_2_O: 517.3214, found: 517.3219.

### Experimental data of 1-benzhydryl-4-(4-(2-((5,6,7,8-tetrahydronaphthalen-2-yl)oxy)ethyl)benzyl)piperazine (18)

White solid (ethyl acetate); M.p. 116.0°C–116.7°C; Yield, 68%. The purity = 98.3%. ^1^H NMR (400 MHz, CDCl_3_): *δ* 7.39 (d, *J* = 7.6 Hz, 4H), 7.23 (dd, *J* = 15.2 Hz, *J* = 7.4 Hz, 8H), 7.17 (t, *J* = 6.6 Hz, 2H), 6.94 (d, *J* = 8.4 Hz, 1H), 6.64 (d, *J* = 8.4 Hz, 1H), 6.59 (s, 1H), 4.22 (s, 1H), 4.10 (t, *J* = 7.1 Hz, 2H), 3.51 (s, 2H), 3.04 (t, *J* = 7.1 Hz, 2H), 2.69 (t, *J* = 5.1 Hz, 4H), 2.49 (s, 4H), 2.43 (s, 4H), 1.75 (d, *J* = 2.6 Hz, 4H). ^13^C NMR (100 MHz, CDCl_3_): *δ* 156.6, 142.7, 138.1, 129.9, 129.5, 129.3, 128.8, 128.4, 128.0, 126.9, 114.5, 112.4, 76.2, 68.7, 62.7, 53.2, 51.7, 35.5, 28.6, 23.4, 23.2. HRMS (ESI) m/z [M + H]^+^: calcd for C_36_H_41_N_2_O: 517.3214, found: 517.3230.

### Experimental data of 1-benzhydryl-4-(4-(2-(o-tolyloxy)ethyl)benzyl)piperazine (19)

White solid (ethyl acetate); M.p. 118.7°C–119.4°C; Yield, 83%. The purity = 98.9%. ^1^H NMR (400 MHz, CDCl_3_): *δ* 7.39 (d, *J* = 7.5 Hz, 4H), 7.25 (s, 2H), 7.22 (d, *J* = 7.2 Hz, 6H), 7.15 (d, *J* = 7.2 Hz, 2H), 7.10 (t, *J* = 7.6 Hz, 2H), 6.82 (t, *J* = 7.4 Hz, 1H), 6.76 (d, *J* = 8.5 Hz, 1H), 4.21 (s, 1H), 4.12 (t, *J* = 6.7 Hz, 2H), 3.50 (s, 2H), 3.06 (t, *J* = 6.7 Hz, 2H), 2.48 (s, 4H), 2.42 (s, 4H), 2.17 (s, 3H). ^13^C NMR (100 MHz, CDCl3): δ 157.0, 142.8, 137.5, 135.8, 130.7, 129.5, 129.0, 128.5, 128.0, 127.7, 126.9, 126.7, 120.3, 110.9, 76.2, 68.7, 62.7, 53.3, 51.8, 35.7, 16.3. HRMS (ESI) m/z [M + H]^+^: calcd for C_33_H_37_N_2_O: 477.2900, found: 477.2911.

### Experimental data of 1-benzhydryl-4-(4-(2-(3,4-dimethylphenoxy)ethyl)benzyl)piperazine (20)

White solid (ethyl acetate); M.p. 126.5°C–127.0°C; Yield, 81%. The purity = 98.4%. ^1^H NMR (400 MHz, CDCl_3_): *δ* 7.37 (d, *J* = 7.1 Hz, 4H), 7.24–7.19 (m, 8H), 7.14 (d, *J* = 7.2 Hz, 2H), 6.99 (d, *J* = 7.8 Hz, 1H), 6.69 (s, 1H), 6.61 (d, *J* = 6.0 Hz, 1H), 4.19 (s, 1H), 4.09 (t, *J* = 7.0 Hz, 2H), 3.52 (s, 2H), 3.02 (t, *J* = 6.9 Hz, 2H), 2.51 (s, 4H), 2.43 (s, 4H), 2.19 (s, 3H), 2.16 (s, 3H). ^13^C NMR (100 MHz, CDCl_3_): *δ* 157.0, 142.8, 137.7, 137.5, 130.4, 130.3, 129.8, 128.9, 128.7, 128.5, 128.0, 126.9, 116.3, 111.6, 76.2, 68.7, 62.6, 53.2, 51.6, 35.6, 20.0, 18.8. HRMS (ESI) m/z [M + H]^+^: calcd for C_34_H_39_N_2_O: 491.3057, found: 491.3067.

### Experimental data of 1-benzhydryl-4-(4-(2-(4-chlorophenoxy)ethyl)benzyl)piperazine (21)

White solid (ethyl acetate); M.p. 129.6°C–130.1°C; Yield, 72%. The purity = 99%. ^1^H NMR (500 MHz, CDCl_3_): *δ* 7.45 (d, *J* = 7.2 Hz, 4H), 7.32 (s, 1H), 7.30 (d, *J* = 6.2 Hz, 5H), 7.26–7.24 (m, 4H), 7.21 (t, *J* = 7.4 Hz, 2H), 6.85 (d, *J* = 9.0 Hz, 2H), 4.28 (s, 1H), 4.15 (t, *J* = 7.1 Hz, 2H), 3.57 (s, 2H), 3.10 (t, *J* = 7.1 Hz, 2H), 2.54 (s, 4H), 2.47 (s, 4H). ^13^C NMR (125 MHz, CDCl_3_): *δ* 157.5, 142.8, 136.8, 136.0, 129.6, 129.3, 128.8, 128.5, 128.0, 126.9, 125.6, 115.9, 76.2, 69.0, 62.6, 53.3, 51.8, 35.4. HRMS (ESI) m/z [M + H]^+^: calcd for C_32_H_34_ClN_2_O: 497.2354, found: 497.2376.

### Experimental data of 1-benzhydryl-4-(4-(2-(naphthalen-1-yloxy)ethyl)benzyl)piperazine (22)

White solid (ethyl acetate); M.p. 110.3°C–110.9°C; Yield, 75%. The purity = 98.5%. ^1^H NMR (500 MHz, CDCl_3_): *δ* 8.31 (d, *J* = 7.2 Hz, 1H), 7.83 (d, *J* = 7.1 Hz, 1H), 7.52–7.49 (m, 2H), 7.46 (d, *J* = 7.1 Hz, 5H), 7.39 (t, *J* = 7.8 Hz, 1H), 7.38–7.29 (m, 8H), 7.22 (t, *J* = 7.4 Hz, 2H), 6.84 (d, *J* = 7.4 Hz, 1H), 4.38 (t, *J* = 6.9 Hz, 2H), 4.29 (s, 1H), 3.57 (s, 2H), 3.27 (t, *J* = 6.9 Hz, 2H), 2.54 (s, 4H), 2.48 (s, 4H). ^13^C NMR (125 MHz, CDCl_3_): *δ* 154.6, 142.8, 137.3, 136.1, 134.5, 129.5, 128.9, 128.5, 128.0, 127.4, 126.9, 126.4, 125.9, 125.7, 125.2, 122.1, 120.3, 104.7, 76.2, 68.9, 62.7, 53.3, 51.9, 35.6. HRMS (ESI) m/z [M + H]^+^: calcd for C_36_H_37_N_2_O: 513.2900, found: 513.2927.

### Experimental data of 1-benzhydryl-4-(4-(2-(4-bromophenoxy)ethyl)benzyl)piperazine (23)

White solid (ethyl acetate); M.p. 127.2°C–127.8°C; Yield, 79%. The purity = 98.6%. ^1^H NMR (500 MHz, CDCl_3_): δ 7.44 (d, J = 7.3 Hz, 4H), 7.38 (dd, J = 7.0 Hz, J = 2.1 Hz, 2H), 7.31 (s, 1H), 7.29 (d, J = 8.0 Hz, 5H), 7.24 (d, J = 8.1 Hz, 2H), 7.20 (t, J = 7.4 Hz, 2H), 6.80 (d, J = 9.0 Hz, 2H), 4.27 (s, 1H), 4.14 (t, J = 7.1 Hz, 2H), 3.55 (s, 2H), 3.09 (t, J = 7.1 Hz, 2H), 2.54 (s, 4H), 2.48 (s, 4H). ^13^C NMR (125 MHz, CDCl_3_): δ 158.0, 142.8, 136.8, 136.0, 132.3, 129.6, 128.9, 128.5, 128.0, 126.9, 116.4, 112.9, 76.2, 69.0, 62.7, 53.3, 51.8, 35.4. HRMS (ESI) m/z [M + H]^+^: calcd for C_32_H_34_BrN_2_O: 541.1849, found: 541.1884.

### Experimental data of 1-benzhydryl-4-(4-(2-(4-(trifluoromethyl)phenoxy)ethyl)benzyl)piperazine (24)

White solid (ethyl acetate); M.p. 109.8°C–110.5°C; Yield, 68%. The purity = 98.7%. ^1^H NMR (500 MHz, CDCl_3_): *δ* 7.57 (d, *J* = 8.7 Hz, 2H), 7.46 (d, *J* = 7.3 Hz, 4H), 7.31 (t, *J* = 7.6 Hz, 6H), 7.27 (d, *J* = 8.0 Hz, 2H), 7.22 (t, *J* = 7.4 Hz, 2H), 6.98 (d, *J* = 8.7 Hz, 2H), 4.30 (s, 1H), 4.22 (t, *J* = 7.1 Hz, 2H), 3.57 (s, 2H), 3.14 (t, *J* = 7.1 Hz, 2H), 2.55 (s, 4H), 2.48 (s, 4H). ^13^C NMR (125 MHz, CDCl_3_): *δ* 161.3, 142.8, 136.6, 136.4, 129.6, 128.9, 128.5, 128.0, 126.9 (t, *J* = 4.6 Hz), 124.5 (dd, *J* = 539.0 Hz, *J* = 269.6 Hz), 122.9 (dd, *J* = 65.0 Hz, *J* = 32.5 Hz), 120.7 (d, *J* = 141.4 Hz), 114.5, 76.3, 68.9, 62.7, 53.4, 51.9, 35.3. HRMS (ESI) m/z [M + H]^+^: calcd for C_33_H_34_F_3_N_2_O: 531.2618, found: 531.2648.

### Biological evaluation

#### *In Vitro* cytotoxic assay

##### Cell culture

PC-3 and WPMY-1 cells were cultured in Dulbecco’s modiffcation Eagle’s medium (DMEM, Invitrogen, Carlsbad, CA, United States) supplemented with 10% fetal bovine serum (FBS, Hyclone, Logan, UT, United States), 100 U/mL penicillin and 0.1 mg/mL streptomycin (Invitrogen). DU145 cells were cultured in RPMI1640 media supplemented with 10% fetal bovine serum (FBS, Hyclone), 100 U/mL penicillin and 0.1 mg/mL streptomycin (Invitrogen). LNCaP cells were cultured in F12 media supplemented with 10% fetal bovine serum (FBS, Hyclone), 100 U/mL penicillin and 0.1 mg/mL streptomycin (Invitrogen). The cells were incubated at 37°C in a humidified atmosphere with 5% CO_2_(Chen et al., 2025; Jiang et al., 2024).

#### Assessment of antitumor activity by CCK-8 assay

The proliferative capacity of cells was evaluated through the implementation of a Cell Counting Kit-8 (CCK-8) assay, supplied by Dojindo (Japan), to quantify cellular growth. Post-transfection, cells were dispensed into a 96-well microplate at a density of 3 × 10^3^ cells per well and incubated for intervals of 0, 24, 48, 72, 96, and 120 h. Following this, 10 μL of the CCK-8 solution was introduced into each well and the plates were returned to the incubator for an additional 2-h period at 37°C under 5% CO_2_ conditions. Absorbance readings at 450 nm were subsequently taken using an ELISA reader manufactured by Bio Tek (United States) (Chen et al., 2022; Zhou et al., 2024).

The compound concentrations were set at 30, 15, 7.5, 3.75, 1.88, and 0.94 μmol/L. The absorbance (A) was measured at 450 nm using a microplate reader. A linear regression analysis was performed plotting the logarithm of compound concentration against the inhibition rate to obtain a straight-line equation from which the half-maximal inhibitory concentration (IC_50_) of the compound, capable of inhibiting 50% of cancer cells, was determined. All experiments were repeated three times under identical conditions, and the mean value was taken as the final result.

#### AR reporter gene assay

Fireffy and Renilla luciferase activities, which are indicated as RLUs, were determined using Dual-Glo luciferase assay kits (Promega) according to the manufacturer’s instructions (Chen et al., 2019a; Chen et al., 2019b). RLUs were measured using a luminometer (GloMaxTM 96-Microplate Luminometer, Promega) and are reported as the mean ± SEM of three individual experiments. For agonists, fold of induction = LUinduced/RLUuninduced. For antagonists, % of control = 100 × RLU (agonist + antagonist)/RLU (agonist alone). All RLUs were normalized against ffreffy RLUs/Renilla RLUs. Data are expressed as EC_50_/IC_50_ values in μM, and the IC_50_ of phenylephrine (μM) was calculated by plotting the data using nonlinear regression analysis in Graph-Pad Prism 5 software.

### Fluorescence polarization (FP)

The fluorescence polarization technique was used to analyze the binding of **7, 11, 17, 19, 20, 21, 22, 23, 24** and enzalutamide to the AR using the PolarScreen™ AR Competitor Assay, Green (lifetechnologies, A15880) according to the manufacturer’s instructions (Chen et al., 2019a; Chen et al., 2019b). Brieffy, the assay entails titration of the test compound against a preformed complex of Fluormone™AL Green and the AR-LBD (GST). The assay mixture was allowed to equilibrate at room temperature in 384-well black plates for 4 h, after which the fluorescence polarization values were measured in a SpectraMax® Paradigm® Multi-Mode Detection Platform (Molecular Devices) using an excitation wavelength of 485 nm and an emission wavelength of 535 nm. Data analysis for the ligand binding assays was performed using Prism software (GraphPad Software, Inc.).

### Molecular docking simulation

Until now, three binding sites of androgen receptor have been reported, including LBP, AF2 and BF3 (Chen et al., 2019a; Chen et al., 2019b). In order to explore the mechanism of androgen receptor antagonism by the target compound **17**, a dockingsimulation was carried out using AutoDock Vina software. The crystal structure of androgen receptor downloaded from the RCSB Protein Data Bank (http://www.rcsb.org/pdb/home/home.do) was taken as the template protein to engage in docking simulation. In prepare, the exogenous ligand was removed and the hydrogen atoms were added to the system. To ensure the reliability of docking simulation, a redock process for the exogenous ligand was performed before docking analysis. Finally, one compound target (i.e., compound **17**) with high AR antagonistic activity was docked into three potential binding sites (including LBP, AF2 and BF3) with 10 configurations.

## Results and discussion

### Chemistry

Scheme 1 illustrated the synthesis of arylpiperazine derivatives **5–24** *via* a four-step reaction starting from 2-(4-(bromomethyl)phenyl)acetic acid. First, 2-(4-(bromomethyl)phenyl)acetic acid one was reduced to alcohol 2 with the presence of a borane–methyl sulfide complex (2 M in tetrahydrofuran) at 0°C for 1 h and at room temperature for 12 h. After the nucleophilic substitution reaction was carried out between compound 2 and 1-(diphenylmethyl)-piperazin using CH_3_CN as solvent in the presence of potassium carbonate at 85°C for 12 h gave 3 (70% yield from 1). Subsequently, compound 4 (87% yield) was obtained by reacting 3 with 4-toluene-sulfonyl chloride using CH_2_Cl_2_ as solvent in the presence of trimethylamine and a catalytic amount of 4-dimethylaminopyridine at 0°C for 16 h. Finally, compound 4 was treated with various phenol (1.5 equiv) in the presence of K_2_CO_3_ (6 eq) to obtain derivatives **5–24** in moderate yields (51%–83%). All synthesized analogs were confirmed using ^1^H-NMR, ^13^C-NMR, and HRMS.

### Cytotoxic activity and AR antagonist activity

The cytotoxic activity of derivatives **5–24** against three human prostate cancer cell lines (PC-3, LNCaP, and DU145) and one type of human normal prostate epithelial cells was evaluated using the CCK-8 method. The results are shown in Table 1.

**TABLE 1:** *In vitro* cytotoxicity of compounds **5–24**.

Compd.	IC_50_ (μM)^a^			
	PC-3^b^	LNCaP^b^	DU145^b^	WPMY-1^b^
**5**	13.08 ± 0.12	>50	>50	>50
**6**	34.39 ± 0.17	>50	>50	>50
**7**	2.95 ± 0.04	>50	24.76 ± 0.21	>50
**8**	12.54 ± 0.11	22.36 ± 0.20	19.83 ± 0.14	17.56 ± 0.12
**9**	15.23 ± 0.19	>50	>50	>50
**10**	17.44 ± 0.07	>50	>50	>50
**11**	1.59 ± 0.02	>50	17.43 ± 0.04	>50
**12**	>50	19.64 ± 0.23	34.43 ± 0.15	28.54 ± 0.16
**13**	15.35 ± 0.08	12.17 ± 0.25	21.24 ± 0.16	17.83 ± 0.17
**14**	19.12 ± 0.16	>50	>50	>50
**15**	20.49 ± 0.12	9.29 ± 0.23	13.69 ± 0.14	14.72 ± 0.14
**16**	13.6 ± 0.09	>50	>50	>50
**17**	1.89 ± 0.14	1.04 ± 0.21	7.32 ± 0.08	>50
**18**	5.02 ± 0.12	>50	14.36 ± 0.15	>50
**19**	1.23 ± 0.15	2.32 ± 0.15	0.87 ± 0.04	>50
**20**	1.05 ± 0.14	2.39 ± 0.13	3.89 ± 0.14	>50
**21**	2.83 ± 0.21	16.09 ± 0.12	13.72 ± 0.14	28.65 ± 0.13
**22**	10.12 ± 0.17	6.78 ± 0.14	15.76 ± 0.09	33.16 ± 0.15
**23**	1.78 ± 0.12	3.86 ± 0.13	1.42 ± 0.11	>50
**24**	3.94 ± 0.14	14.58 ± 0.18	5.76 ± 0.14	>50
Naftopidil	42.10 ± 0.79	22.36 ± 0.61	34.58 ± 0.31	>50

^a^ IC_50_ refers to the half-maximal inhibitory concentration. The IC_50_ values were calculated as the mean ± standard deviation from three experimental trials.

^b^ PC-3, LNCaP, and DU145 are human prostate cancer cell lines; WPMY-1, refers to human normal prostatic epithelial cells.

In this study, the cytotoxic activity of ether arylpiperazine derivatives ranging from **5** to **24** was assessed against 4 cell lines: PC-3, LNCaP, DU145 (all human prostate cancer cell lines), and WPMY-1 (human normal prostatic epithelial cells) using the CCK-8 assay. The outcomes are summarized in Table 1. The data presented in the table reveal that several of these compounds exhibit pronounced cytotoxic activity against the tested cancer cell lines, with some demonstrating significantly greater potency compared to naftopidil. Notably: Compounds **7, 11, 17, 19, 20, 21, 23**, and **24** exhibit exceptionally potent activity against PC-3 cells, characterized by IC_50_ values below 5 μM. For LNCaP cells, compounds **17, 19, 20**, and **23** show particularly strong activity with IC_50_ values less than 5 μM. Compounds **19, 20, 23** and **24** also demonstrate pronounced cytotoxicity against DU145 cells. Moreover, the majority of compounds exhibited low cytotoxic character toward normal human prostate epithelial cells (WPMY-1).

**FIGURE 2:**
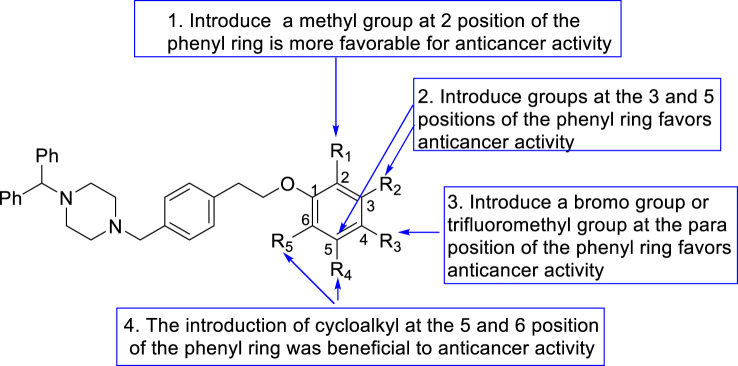
The SAR summary of the derivatives **5**–**24**.

The SAR investigation was mainly focused on the variation of the substitute’s type on the phenyl group as a required group for antitumor activity. (1) For instance, compared to phenylpiperazine compound **5**, compounds **7** (IC_50_ = 2.95 μM) and **11** (IC_50_ = 1.59 μM), which introduce two symmetrical groups onto the phenyl ring, exhibit potent cytotoxic activity against PC-3 cells. In contrast, compound **12** (IC_50_ > 50 μM), featuring a methyl group as an electron-donating substituent in the meta position of the phenyl ring, displays significantly weaker cytotoxic activity against PC-3 than **5**. Additionally, compound **24** (IC_50_ = 5.76 μM), with a trifluoromethyl group as a strong electron-withdrawing substituent on the phenyl ring, exhibits strong cytotoxic activity against DU145 cells. These activity results indicate that variations in substituents on the phenyl ring do have a certain impact on cytotoxic activity. (2) A comparison between compounds **10** and **11** reveals that compound **11** (IC_50_ = 1.59 μM) demonstrates exceptionally strong activity against PC-3 cells, while compound **10** exhibits weaker cytotoxic activity against PC-3. These activity results suggest that the introduction of symmetrical, weakly electron-withdrawing substituents at the 3 and 5 positions of the phenyl ring favors anticancer activity. (3) When comparing compound **19** with compounds **13** and **14**, compound **19** (IC_50_ = 0.87 μM) exhibits more pronounced cytotoxic activity against DU145 cells. The results indicate that the introduction of electron-donating groups in the ortho position of the phenyl ring is more favorable for anticancer activity than the introduction of electron-withdrawing groups. (4) A comparison between compounds **12, 15**, and **19** reveals that compound **19** (IC_50_ > 50 μM) exhibits weak activity against human normal prostatic epithelial cells. These activity results suggest that when introducing a methyl group at different positions on the phenyl ring, the ortho position favors anticancer activity more than other positions. (5) Comparing compound **7** with compound **12**, compound **7** (IC_50_ = 2.95 μM) demonstrates more pronounced cytotoxic activity against PC-3 cells. These activity results indicate that the simultaneous introduction of methyl groups at the three and five positions of the phenyl ring favors anticancer activity more than the introduction of a methyl group only at the three position. (6) When comparing compounds 6, 8, 9, 15 and 21, compounds 23 and 24 exhibits more versatile and superior cytotoxic activity. These activity results suggest that the introduction of a bromo group or a trifluoromethyl group at the para position of the phenyl ring favors anticancer activity. (7) A comparison between compounds 17 and 18, compound 17 (IC50 = 1.89 μM, 1.04 μM, 7.32 μM) demonstrates better activity against the tested cell lines than compound 18 (IC50 = 5.02 μM, >50 μM, 14.36 μM). The results showed that the introduction of cycloalkyl at the 5 and 6 position of the phenyl ring was beneficial to anticancer activity (Figure 2). Above results can lead to a tool which can further design arylpiperazine derivatives as AR antagonists for *in vitro* and *in vivo* studies.

To further investigate whether these derivatives possess antagonistic activity against AR, this study adopted the scientific method of luciferase assay (Qi et al., 2022a; Qi et al., 2022b) to more accurately evaluate the antagonistic effects of these derivatives on AR (Table 2). During the implementation of the AR luciferase assay experiments, we specifically added 1 nM of the AR agonist R1881 for co-treatment, and quantitatively assessed the strength of the antagonistic activity based on the degree of inhibition of luciferase expression induced by R1881. According to the data presented in Table 2, it is evident that compounds **7, 11, 21, 22**, and **24** exhibit relatively weak antagonistic effects on AR. However, notably, compounds **17, 19, 20**, and **23** demonstrate significant antagonistic efficacy, with inhibition rates exceeding 55%. It is worth noting that these results do not entirely align with previous tests on anti-proliferative activity against cancer cells. These findings suggest that the skillful introduction of certain small molecular groups on the piperazine ring may significantly enhance their antagonistic activity against AR. The conclusions drawn from this study undoubtedly provide us with a powerful tool, which will aid us in delving deeper into the interaction mechanisms between piperazine derivatives and AR, thus laying a more solid theoretical foundation for future drug development.

**TABLE 2:** AR antagonist activity of compounds.

Compound	AR antagonistic activity% (10 μM)^a^
**7**	50.1 ± 1.1
**11**	51.2 ± 1.3
**17**	70.3 ± 0.8
**19**	71.5 ± 0.5
**20**	65.7 ± 1.4
**21**	49.4 ± 0.8
**22**	40.3 ± 1.2
**23**	62.2 ± 1.5
**24**	47.3 ± 0.6
**R1881**	N.E^b^
**Naftopidil**	52.2 ± 0.3
**Enzalutamide**	84.7 ± 1.4

^a^ Inhibition rate was shown as a ratio to the R1881 control.

^b^ N.E, no antagonistic effect.

Recent studies have shown that, in addition to the AR signaling pathway, estrogen receptors (ER) also play an important role in the pathogenesis of PCa(Belluti et al., 2023; Souza et al., 2023). Research by Sarswat et al. (Sarswat et al., 2011) discovered that arylpiperazines, in addition to inhibiting the transmission of AR signals, can also promote the expression of ER-β in prostate cancer cells. ER-β may function as a protective receptor, exerting inhibitory effects on the development and malignant progression of PCa(Chaurasiya et al., 2020; Lombardi et al., 2020). By acting on prostate cancer cells through these two signaling pathways simultaneously, arylpiperazines inhibit their proliferation.

### Binding affinity assay of compounds 7, 11, 17, 19, 20, 21, 22, 23 and 24

To delve into the specific binding characteristics of these analogs that exhibit significant inhibitory activity against the AR, we conducted detailed binding affinity studies using fluorescence polarization (FP) technology (Chen et al., 2023; He et al., 2021; Xue et al., 2023). The experimental design was based on the competitive binding mechanism between fluorescent tracers and non-fluorescent antagonists, aiming to assess the interaction strength between a series of compounds numbered **7, 11, 17, 19, 20, 21, 22, 23** and **24** with AR (Table 3). Through this method, we were able to precisely measure the binding efficiency of each compound at different concentrations and summarize the results in Table 3. The study revealed that all tested analogs demonstrated strong binding affinity to AR, with IC_50_ values below 4 μmol (μM), indicating high binding affinity. Particularly noteworthy, compounds **17** and **19** exhibited outstanding binding performance, with IC_50_ values of 1.14 μM and 1.01 μM, respectively, surpassing not only other test samples but also the clinically used standard drug enzalutamide (IC_50_ = 2.56 μM). This suggests that these two compounds may serve as more effective candidates for AR antagonists.

**TABLE 3:** The binding affinity of compounds to mutant AR.

Compound	IC_50_/µm^a^
**7**	2.15 ± 0.13
**11**	2.01 ± 0.15
**17**	1.14 ± 0.12
**19**	1.01 ± 0.03
**20**	1.66 ± 0.12
**21**	2.74 ± 0.16
**22**	3.02 ± 0.24
**23**	1.72 ± 0.12
**24**	2.87 ± 0.22
Naftopidil	2.13 ± 0.07
Enzalutamide	2.56 ± 0.27

^a^ The data represent the mean of at least three independent determinations.

Further analysis revealed a clear trend among the tested arylpiperazine derivatives: a direct correlation between the binding affinity of compounds to AR and their antagonistic activity. For instance, the tightly bound compounds **17** and **19** were also the most effective antagonists, achieving maximum inhibition rates of 70.3% and 71.5%, respectively. Additionally, while not as prominent as the former two, compounds **20** and **23** also exhibited strong antagonistic effects, with inhibition rates of 65.7% and 62.2%, respectively. These observations support the hypothesis that enhanced binding affinity may be a key factor in improving AR antagonistic activity. Based on these findings, it can be inferred that certain ether-substituted arylpiperazine derivatives, due to their excellent binding ability and antagonistic efficacy, hold potential for development as novel AR antagonists, especially in the field of prostate cancer treatment. Considering the exceptional characteristics displayed by compound **19**, we have decided to focus on it for the next phase of research to explore its specific binding sites with AR and potential mechanisms of action. This will contribute to understanding how these compounds effectively block the AR signaling pathway, providing new strategies and methods for the treatment of prostate cancer.

### Docking study

To decipher the binding mode of these compounds, as well as to explore the detailed information about their major binding interactions with AR (Zhang et al., 2014) docking simulation is performed. The optimal antagonist, compound **19** was taken as the template molecule in this process, and three binding sites of AR, including ligand binding pocket (LBP), activation function-2 (AF2) and binding function 3 (BF3) (Axerio-Cilies et al., 2011; Lack et al., 2011), were all used to explore the binding affinities of this compound. The lowest docked energy values were summarized in Table 3.

**FIGURE 3:**
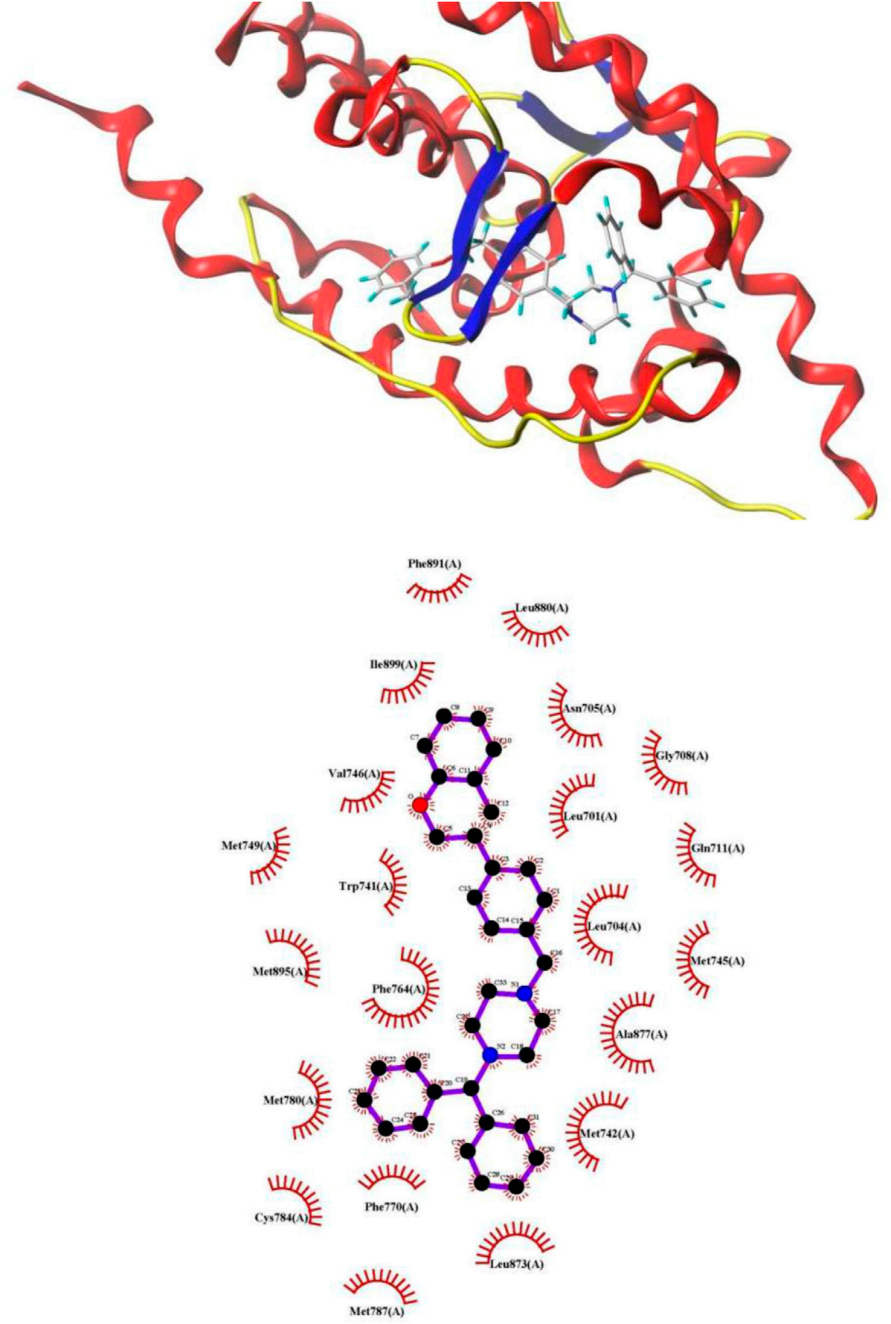
The view of compound **19**-AR interaction plots.

As shown in Table 4, LBP binding site had the highest binding force of −11.01 kcal/mol, which indicated that AR LBP was the major binding site for compound **19**. To better discover these binding interactions, the binding mode of compound **19** with AR was analyzed, and the detailed information was displayed in Figure 3. As shown in Figure 3, compound **19** could fit into the AR LBP site by the formation of Van der Waals’ force with 14 amino acid residues, such as Val 746, Trp 741 and Phe 764, and so on. These results showed that the compound **19** mainly bind to AR LBP site through the interaction of Van der Waals’ force.

**TABLE 4:** The binding affinities (kcal/mol) of compound **19** in three binding sites of AR.

Binding site	Compound 19	Naftopidil
LBP (PDB ID: 2OZ7)	−11.01	−7.29
AF2 (PDB ID: 2YHD)	−4.86	−6.87
BF3 (PDB ID: 2YLO)	−6.86	−8.1

## Conclusion

In conclusion, this study reported the synthesis and biological evaluation of a series of novel arylpiperazine derivatives against three human prostate cancer cells and human prostate epithelial cells and AR, respectively. The results showed that the derivatives **7, 11, 17, 19, 20, 21, 22, 23** and **24** displayed strong cytotoxic activities against the tested cancer cells, and derivatives **17, 19, 20**, and **23** exhibited relatively strong antagonistic potency against AR (Inhibition% >60) and exhibited potent AR binding affinities. Structure-activity relationship (SAR) studies indicated that the introduction of cycloalkyl groups at the m,p-position on the phenyl ring and a methyl group at the o-position on the phenyl ring favored enhanced activity. Docking study suggested that the compounds **19** mainly bind to AR ligand binding pocket (LBP) site through the interaction of Van der Waals’ force. These piperazine derivatives may guide the structural modification of novel anti-prostate cancer drugs.

## Data Availability

The datasets presented in this study can be found in online repositories. The names of the repository/repositories and accession number(s) can be found in the article/Supplementary Material.
