# Supplementary material for: Synthesis, bioactivity, and molecular docking studies: novel arylpiperazine derivatives as potential new-resistant AR antagonists
Source: Front Chem. 2025 Mar 28;13:1557275. doi: 10.3389/fchem.2025.1557275 (PMC11985751; doi:10.3389/fchem.2025.1557275)
Supplement: Supplementary file 1 [file DataSheet1.docx]

**Supporting information**

**Synthesis, bioactivity, and molecular docking studies: novel arylpiperazine derivatives as potential new-resistant AR antagonists**

Hua Jiang^1,a^[[1]](#footnote-0)^*^, Haowei Chen^2,a^, Ya Wang^2^, Huaxin Xu^2^, Hong Chen^2^[[2]](#footnote-1)^*^

*1 Department of Urology, the Fifth Affiliated Hospital of Zunyi Medical University (Zhuhai Sixth People's Hospital), Zhuhai, 519100, China*

*2 Luoyang Key Laboratory of Organic Functional Molecules, College of Food and Drug, Luoyang Normal University, Luoyang, 471934, China*

Reagents and solvents were procured via commercial channels. Organic solvents underwent distillation before use. Melting points were determined using an uncalibrated SGW X-4 micro melting point apparatus. NMR spectra were acquired on a Bruker AVANCE-400 spectrometer in CDCl_3_, employing TMS as an internal standard, with chemical shifts reported in δ (ppm) and coupling constants in Hertz. HRMS spectra were documented on an AB Sciex X500R QTOF mass spectrometer (Foster City, California, USA). The completion of all reactions was monitored by thin-layer chromatography (TLC) performed on pre-coated silica gel 60 F_254_ TLC plates (VWR), with observations made under ultraviolet light at wavelengths of 254 and/or 365 nm.

Scheme 1. The synthesis route of derivatives 5–24.

Reagents and conditions: (i) BH3·S(CH3)2, THF, 0℃ for 1 h, and then room temperature for 10 h; (ii) 1-(Diphenylmethyl) piperazine, K_2_CO_3_, CH_3_CN, reflux, 12 h; (iii) TsCl, Et_3_N and 4-dimethylaminopyridine, Cl_2_CH_2_, 0℃, 16 h; (iv) Phenol, K_2_CO_3_, CH_3_CN, reflux, 12 h.

**The NMR spectra (^1^H NMR, ^13^C NMR) of compounds 5-24**

**^1^H NMR of compound 5**

**^13^C NMR of compound 5**

**^1^H NMR of compound 6**

**^13^C NMR of compound 6**

**^1^H NMR of compound 7**

**^13^C NMR of compound 7**

**^1^H NMR of compound 8**

**^13^C NMR of compound 8**

**^1^H NMR of compound 9**

**^13^C NMR of compound 9**

**^1^H NMR of compound 10**

**^13^C NMR of compound 10**

**^1^H NMR of compound 11**

**^13^C NMR of compound 11**

**^1^H NMR of compound 12**

**^13^C NMR of compound 12**

**^1^H NMR of compound 13**

**^13^C NMR of compound 13**

**^1^H NMR of compound 14**

**^13^C NMR of compound 14**

**^1^H NMR of compound 15**

**^13^C NMR of compound 15**

**^1^H NMR of compound 16**

**^13^C NMR of compound 16**

**^1^H NMR of compound 17**

**^13^C NMR of compound 17**

**^1^H NMR of compound 18**

**^13^C NMR of compound 18**

**^1^H NMR of compound 19**

**^13^C NMR of compound 19**

**^1^H NMR of compound 20**

**^13^C NMR of compound 20**

**^1^H NMR of compound 21**

**^13^C NMR of compound 21**

**^1^H NMR of compound 22**

**^13^C NMR of compound 22**

**^1^H NMR of compound 23**

**^13^C NMR of compound 23**

**^1^H NMR of compound 24**

**^13^C NMR of compound 24**

| **Primer for PCR** | |
| --- | --- |
| Primer Name | Sequence (5'-3') |
| GAPDH-F | GGGAAACTGTGGCGTGAT |
| GAPDH-R | GAGTGGGTGTCGCTGTTGA |
| AR-F | ACTCTGGCTTCACAGTTTGGA |
| AR-R | TTCCCTTCAGCGGCTCTTTT |

**Supplemental Table S1.** The sequence of primers used for experiments in this study.

Methods: The quantitative real time PCR (qRT-PCR) analysis was carried out using SYBR Premix Ex TaqⅡ (Takara Bio Inc.) with 7500 Real-Time PCR system (Applied Biosystems) according to the manufacturer's protocol. Glyceraldehyde 3-Phosphate Dehydrogenase (GAPDH) expression was used as an internal control. For quantitative results, the relative expression level of AR and GAPDH were calculated via the 2^−ΔΔCt^ calculation.


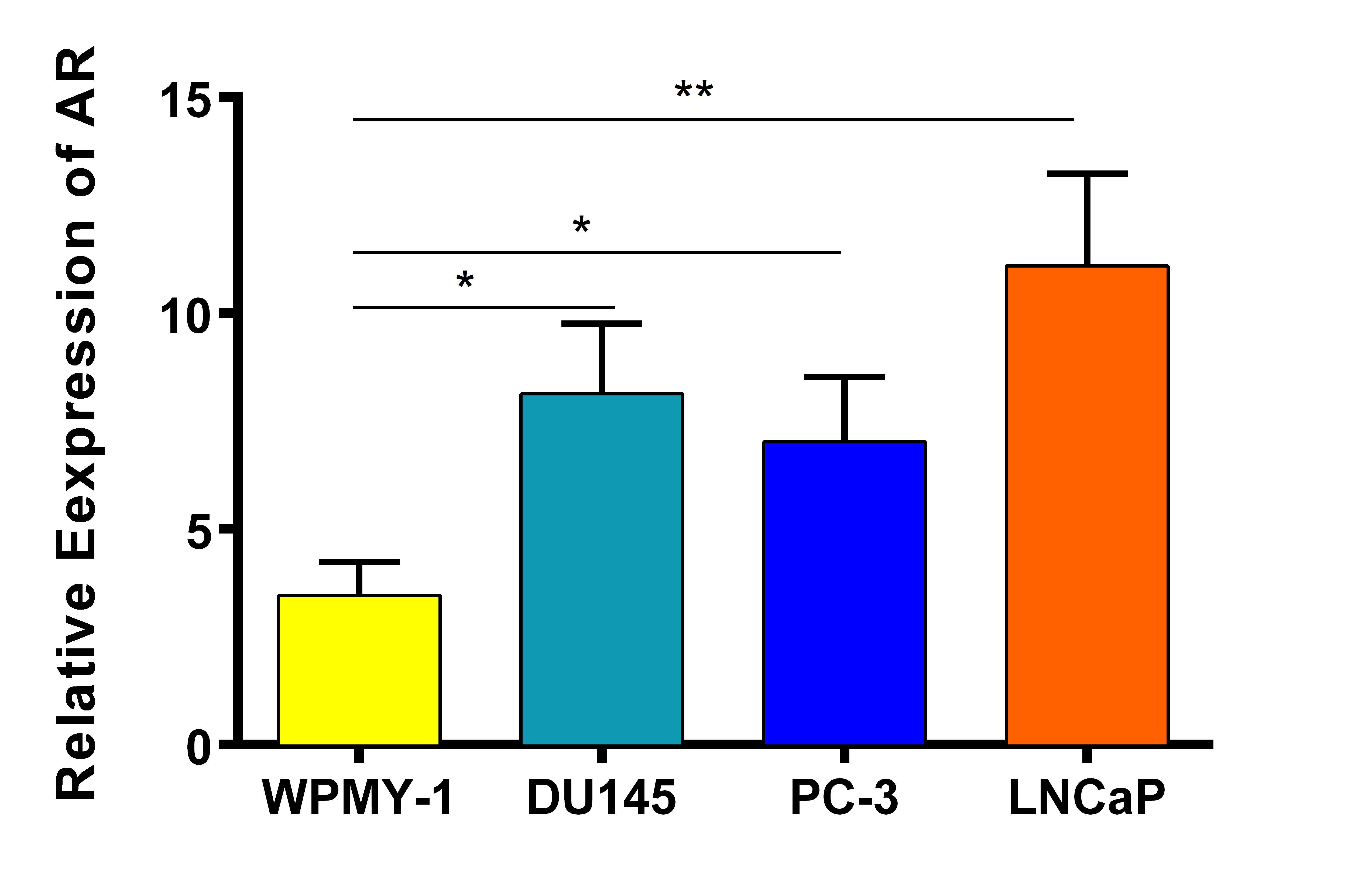


1. ^*^ Correspondence: yonghongjiang@163.com (H. J.); chenwexpo@163.com (H. C.)

   ^a^ Both authors contributed equally to the work. [↑](#footnote-ref-0)
2. [↑](#footnote-ref-1)
